# *LRRK2* mutant knock-in mouse models: therapeutic relevance in Parkinson's disease

**DOI:** 10.1186/s40035-022-00285-2

**Published:** 2022-02-14

**Authors:** Eunice Eun Seo Chang, Philip Wing-Lok Ho, Hui-Fang Liu, Shirley Yin-Yu Pang, Chi-Ting Leung, Yasine Malki, Zoe Yuen-Kiu Choi, David Boyer Ramsden, Shu-Leong Ho

**Affiliations:** 1 Division of Neurology, Department of Medicine, Queen Mary Hospital, University of Hong Kong, Pok Fu Lam, Hong Kong China; 2 Institute of Metabolism and Systems Research, University of Birmingham, Birmingham, UK

**Keywords:** Parkinson’s disease, LRRK2, Knock-in mouse model, Neurotransmission, Motor dysfunction, Autophagy, Lysosome, Mitochondrial dysfunction, Synucleinopathy, Hyperkinase activity, LRRK2 inhibitor

## Abstract

Mutations in the leucine-rich repeat kinase 2 gene (*LRRK2*) are one of the most frequent genetic causes of both familial and sporadic Parkinson’s disease (PD). Mounting evidence has demonstrated pathological similarities between *LRRK2*-associated PD (*LRRK2*-PD) and sporadic PD, suggesting that LRRK2 is a potential disease modulator and a therapeutic target in PD. *LRRK2* mutant knock-in (KI) mouse models display subtle alterations in pathological aspects that mirror early-stage PD, including increased susceptibility of nigrostriatal neurotransmission, development of motor and non-motor symptoms, mitochondrial and autophagy-lysosomal defects and synucleinopathies. This review provides a rationale for the use of *LRRK2* KI mice to investigate the LRRK2-mediated pathogenesis of PD and implications from current findings from different *LRRK2* KI mouse models, and ultimately discusses the therapeutic potentials against LRRK2-associated pathologies in PD.

## Background

Parkinson’s disease (PD) is the second most common neurodegenerative disorder only next to Alzheimer’s dementia, affecting 1%–2% of the world population aged 65 or above [1]. PD is best distinguished from other neurodegenerative diseases by its characteristic progressive motor symptoms including bradykinesia, dyskinesia, tremor, rigidity and postural instability, which usually occur together with other non-motor symptoms including cognitive impairment, dementia, olfactory dysfunction, depression and rapid eye movement behavioural sleeping disorder [2–4]. Pathophysiological changes in PD involve selective neurodegeneration of neuromelanin-containing dopaminergic neurons in the substantia nigra pars compacta (SNpc) and the formation of insoluble protein aggregates in midbrain termed Lewy bodies (LB) and Lewy neurites, which are mainly composed of α-synuclein [5, 6]. By the time PD motor symptoms emerge, a major portion of the dopaminergic population and its projection into the dorsal striatum have already degenerated. Current therapies for PD including levodopa and dopamine (DA) agonists can effectively alleviate motor symptoms, yet their efficacy diminishes over time and fails to prevent the disease progression.

Despite its clinical documentation in 1817 by Dr. James Parkinson, the exact etiology and pathogenesis underlying PD remain unknown. Although PD is still largely a sporadic disease, approximately 5%–10% of the cases are attributable to genetic causes [7]. Thus far, genome-wide association studies (GWAS) have identified a number of gene variants conferring a risk of PD, which have been discussed in-depth in other reviews [8, 9]. Among the identified PD-causing genetic factors, mutations in the leucine-rich repeat kinase 2 gene (*LRRK2*), located in the *PARK8* locus of chromosome 12q12 [10], cause autosomal dominant form of PD and account for the majority of all known heritable PD [11, 12].

The *LRRK2*-PD cases appear clinically and symptomatically indistinguishable from those of idiopathic PD [13–15], suggesting that LRRK2 may also contribute to the pathogenesis of idiopathic PD. Moreover, several meta-analyses of GWAS have indicated that *LRRK2* is a potent disease-risk locus that is associated with increased risk of developing sporadic PD [16, 17]. Neuropathologically, *LRRK2*-PD cases are highly heterogeneous. Upon autopsy, some *LRRK2*-PD patients possess the classic LB pathology and dopaminergic neuronal loss in the substantia nigra [18], whereas some others display nigral neurodegeneration without any protein aggregate pathology [19, 20]. Other diverging pathologies include the accumulation of tau neurofibrillary tangles or the presence of cytoplasmic inclusions in glial cells [21]. Even different individuals with *LRRK2*-PD within the same family show pleomorphic neuropathology [10, 22]. Nevertheless, DA neurodegeneration in the SNpc region remains the common pathology observed in all *LRRK2*-PD cases, adding weight to the notion that LRRK2 may be involved in some convergent pathways shared by *LRRK2*-PD and sporadic PD. Therefore, *LRRK2*-based animal models may not only be useful for studies of *LRRK2*-PD, but also be applicable to a wider spectrum of this disease.

PD is characterized by progressive neurodegeneration of DA neurons, which leads to various motor symptoms and the presence of α-synuclein pathology in distinct brain regions. To date, no single animal model of PD can faithfully recapitulate both the neuropathological development and the motor symptoms of PD. Nevertheless, progress has been made to elucidate the cellular molecular pathways implicated in PD using different genetic-based cellular and animal models.

As PD is an aging-related disease, various applicable organisms such as yeast, roundworms, zebrafish, *Drosophila* and rodents have been developed as *in vivo* models for studying PD [23, 24]. Whereas most of these models have shown robust phenotypes relevant to PD, each model has technical limitations, such as differences in lifespan, genetic sequence homology, neuroanatomical structure and the complexity of motor and non-motor behaviours. The characteristic neuropathological hallmark of PD is DA neuronal loss and the resulting locomotor defects. In this aspect, mouse models in particular, are one of the most frequently used experimental platforms because of their neuroanatomical similarities to humans. The mouse brain possesses a nigrostriatal system with dopaminergic pathways, dopaminergic alterations in which are reflected in subsequent changes of animal behaviour, potentially mirroring the defective dopaminergic pathway-mediated motor symptoms in human PD [24–26]. Moreover, the mouse genome has an overall 90% similarity to the human genome [27] and a murine homolog of *LRRK2,* which is up to 88% identical to human *LRRK2* in sequence, showing conservation of PD-causing residues [28, 29]. Having an approximately two-year lifespan and an ability to give rise to a reasonable number of offspring, mice have become a valuable and convenient model for experimentation with the help of genetic engineering. In addition, various genetic lines of mouse models can be further cross-bred to develop other transgenic lines of mouse models to fit different experimental objectives and allow target-oriented research into PD.

The development of *LRRK2*-PD animal models in the last two decades has greatly advanced our understanding of the role of LRRK2 in PD neuropathogenesis. In this review, we will first give a brief overview of LRRK2 biology and its relevance in PD, followed by comparative discussion on currently available LRRK2-based mouse models, with a particular focus on *LRRK2* knock-in (KI) mouse models, and several neuropathological and phenotypic aspects as they relate to PD, including striatal neurotransmission, locomotor function, mitochondrial dysfunction, autophagy-lysosomal pathway and synucleinopathies. This review also addresses the current progress on LRRK2 as a potential therapeutic target, and ways by which the *LRRK2* KI mouse model may facilitate the development of therapeutics in PD.

## Main text

### LRRK2

Since the first discovery of LRRK2, much effort has been made to decipher the structure and biological functions of this protein. LRRK2 is a large 286-kDa, multi-domain protein composed of four protein–protein interacting domains: armadillo, ankyrin, leucine-rich repeats (LRR) and WD40, and three enzymatic domains including a kinase domain which confers kinase activity, and the Ras of complex (Roc) and Carboxy terminal of Roc (COR) tandem domains which confer GTPase activity [30]. Functionally, LRRK2 is known to play a key role in several cellular processes, including endo-lysosomal vesicle trafficking [31–33], mitochondrial homeostasis [34], autophagy [35], neurite outgrowth [36, 37], cytoskeletal maintenance [38, 39], and immune system function [40], some of which are relevant to PD and will be discussed in later sections.

LRRK2 is a serine-threonine kinase that is capable of undergoing autophosphorylation [41] and phosphorylation of several downstream substrates such as endophilin A [42], snapin [42], synaptojanin-1 [43] and a subset of Rab GTPases [44]. The Rabs (Rab3A/B/C/D, Rab8A/B, Rab10, Rab12, Rab29, Rab35, and Rab43), which are more recently identified to be physiological substrates of LRRK2 [44], have been frequently employed in both *in vitro* and *in vivo* studies as established readout markers of LRRK2 kinase activity*.* The GTPase activity (or GTP hydrolysis) of LRRK2 is mediated by the highly conserved Roc-COR tandem domain. The exact mechanism of LRRK2 GTPase activity is still under debate, yet some data from prokaryotic Roc-COR domains suggest that LRRK2 may be a GAD (G proteins activated by guanine nucleotide-dependent dimerization) enzyme, which requires dimerization for GTPase activity [45]. Another study shows that the COR domain may serve as the element within the protein’s structure that enables homo-dimerization to occur [46, 47], further supporting the LRRK2 dimerization model. A growing body of evidence suggests that LRRK2 cycles between monomeric and dimeric forms in a GTP/GDP-dependent manner, which influences both its activation and subcellular localization. The monomeric LRRK2 resides mainly in the cytosol and exhibits low kinase activity, and is deemed to be an “inactive” form [48]. LRRK2 dimers display an enhanced kinase activity compared to its monomer and are substantially enriched at the membranes of various organelles, including microsomes, synaptic vesicles, lysosomes and mitochondria [49–51]. The activation of LRRK2 kinase activity is also regulated in part by one of its kinase substrates, Rab29, which recruits LRRK2 to the Golgi complex and promotes its dimerization [52, 53].

Members of the family of highly conserved regulatory proteins 14-3-3 have also been shown to bind to LRRK2 and modulate its localization *via* protein kinase A (PKA)-mediated phosphorylation of several residues of LRRK2, including Ser910 and Ser935 of the N-terminus and Ser1444 within the Roc domain [54, 55]. The PKA-mediated 14-3-3 binding to LRRK2 has been proposed to negatively regulate the kinase activity of LRRK2 by stabilizing an inactive conformation of LRRK2 [55]. The 14-3-3 binding is missing in several pathogenic LRRK2 mutations, such as R1441G, Y1699C and I2020T [54], which is associated with aberrant hyperactive kinase activity of LRRK2. Therefore, the activation of LRRK2 is influenced by its dimerization, GTPase activity, and interaction with other regulatory proteins. Nevertheless, it is unclear whether other protein–protein interacting domains of LRRK2 contribute to its activation and further studies are needed to reveal a more complete picture.

In humans, LRRK2 is expressed in various organs including the lungs, heart, liver and kidneys, and at a lower level throughout the brain, including caudate and putamen of dorsal striatum which receive dopaminergic innervation [11, 56, 57]. LRRK2 is also highly expressed in immune cells, with enhanced expression in monocytes and T-cells of PD patients, implicating its role in the immune system. Similarly, studies in rodent tissues have shown that Lrrk2 is expressed in various tissues including the lungs, kidneys, spleen, heart and immune cells such as microglia and astrocytes [57–60]. Lrrk2 is also highly expressed in the central nervous system of mice, particularly in the putamen of striatum, in which Lrrk2 expression levels surge drastically after birth [61]. In humans, temporal and spatial characterization of LRRK2 expression, particularly in the brain, is lacking due to the limited samples availability [60]. Nevertheless, the patterns of LRRK2 expression appear to be comparable in both humans [56] and mice [61], including its expression in the dopaminoceptive brain areas implicated in PD.

### *LRRK2* mutations in PD

Two years after the first mapping of the PARK8 locus which segregates with PD in 2002 [62], the gene which is mutated in PARK8—later termed *LRRK2*—was identified by two independent groups [10, 11]. Up to date, a number of missense mutations in *LRRK2* have been reported and associated with PD [63, 64], with eight protein variants confirmed to be pathogenic (N1437H, R1441C/G/H/S, Y1699C, G2019S, and I2020T) [16, 65]. Epidemiologically, pathogenic variants of LRRK2 display varying frequencies of expression in different regions or specific populations. For instance, the G2019S variant may account for up to 40% of the Arab descents [66] and approximately 20% of Ashkenazi Jewish population [67], yet it is rarely seen in Asian populations (i.e. Chinese, Japanese, Korean and Indian) [12, 68]. Conversely, the R1441G variant is found most frequently in the Basque region of Spain [69, 70] and the Y1699C variant is found mainly in British kindred [71]. Other non-causative variants, such as R1628P and G2385R located in COR and WD40 domains, respectively, are associated with an increased risk of PD and are more commonly found in Han Chinese and East Asians [72]. Thus, the prevalence of different LRRK2 variants seems to be population-specific, an aspect to be considered when screening for PD mutations.

LRRK2 variants also have a variable rate of lifetime penetrance. Although G2019S is the most frequently occurring mutation, accounting for nearly 4% of all hereditary PD cases and 1% of sporadic PD [12], its penetrance is relatively low ‒ being 24% at 75 years, compared to the 95% disease penetrance of R1441G variant at the same age [73]. Nevertheless, one study noted no difference in age at onset and clinical features among sporadic PD patients and PD patients carrying either the R1441C or the G2019S variant, indicating that the pathogenicity of different LRRK2 mutations in different domains may produce comparable disease phenotypes to those of sporadic PD patients [73].

Among the different domains of LRRK2, much attention has been given to the kinase and Roc-COR domains, due to the presence of major pathogenic variants of PD that alter enzymatic activities. Pathogenic variants G2019S and I2020T are located within the kinase domain and confer increased kinase activity, as demonstrated by their increased autophosphorylation levels [28, 74] and their interaction with several downstream heterologous substrates [28, 44, 75], whereas the R1441C/G/H/S and Y1699C variants within the Roc-COR domains are associated with reduced GTP hydrolysis [74, 76, 77]. This is in line with the finding that R1398H mutation in the Roc domain with enhanced GTP hydrolysis and reduced GTP binding is a protective variant against PD [78, 79].

Interestingly, several pathogenic mutations in Roc-COR domains have also been shown to increase LRRK2 autophosphorylation [44, 74, 80, 81]. In particular, variants such as R1441C/G phosphorylate Rab8 and Rab10 to an even greater extent than the G2019S variant located in the kinase domain [44]. Conformationally, Roc GTPase and kinase domains are assumed to be in close spatial proximity [82]. Therefore, it is not surprising that the GTP binding in the ROC domain could somehow regulate the LRRK2 kinase activity [83, 84]. Whether these two key catalytically active domains of LRRK2 act in converging disease pathways or mediate their actions independently requires further validation. A more comprehensive review on different LRRK2 mutations and their potential interaction can be found elsewhere [85].

Nevertheless, the majority of PD-linked pathogenic mutations lead to increased kinase activity *in vitro* and *in vivo* [41, 44], suggesting that the abnormal enzymatic activities of the different variant forms of LRRK2 may contribute to PD pathogenesis. A growing body of evidence suggests that these hyperactive variants of LRRK2 are associated with a toxic gain-of-function effect [10, 11, 86], possibly through hyperphosphorylation of its downstream substrates, including Rab GTPases [44]. Many of the LRRK2 substrates play a role in regulating mitochondrial dynamics, synaptic vesicle trafficking and the autophagy-lysosomal system. Defects in such cellular pathways are closely associated with PD [87–90], implicating a pathogenic role of LRRK2 variant in PD. Recent evidence also shows that the SNpc of idiopathic PD patients display increased kinase activity of LRRK2 [91] and analysis of urine exosomes isolated from idiopathic PD patients showed an increased level of LRRK2 auto-phosphorylation at the Ser1292 residue [92]. These data support the notion that the pathogenic hyperactive LRRK2 may play a role in overall progression of familial and sporadic PD. This renders LRRK2 as one of the potential therapeutic targets against not only LRRK2-associated PD but a wider spectrum of the disease.

### *LRRK2*-based mouse models

Since the discovery of LRRK2 gene as a causative factor for the autosomal dominant form of PD as well as sporadic PD [62], various LRRK2 mouse models have been developed, including (1) *LRRK2* knockout (KO), (2) *LRRK2* knock-down (KD), (3) overexpression of wildtype (WT) or variant *LRRK2*, and (4) KI of *LRRK2* variants, for elucidating LRRK2 biology and pathophysiology. Below, we will outline PD-relevant phenotypes observed from studies on LRRK2 transgenic, KO and KI mouse models.

#### *LRRK2* KO mice

Mouse models lacking *Lrrk2* gene expression have been generated to study the functional role of LRRK2 in PD pathogenesis. Despite the fact that LRRK2 mutation is one of the key genetic risks of PD, *Lrrk2* KO mice do not recapitulate characteristics of *LRRK2*-PD in humans, with only modest to no locomotor symptoms without dopaminergic neuronal cell loss [93–96]. In contrast to *Lrrk2* KO animals, however, mice having double KO of both *Lrrk2* and its functional homologue *Lrrk1* surprisingly show age-dependent dopaminergic neurodegeneration [97]. The authors suggest that such differences may be due to a compensatory role of LRRK1 in the absence of LRRK2. The phenotype observed in double-KO mice highlights a potential role of LRRK in survival of dopaminergic neurons, although the exact mechanistic interaction between LRRK1 and LRRK2 is not fully studied. Nevertheless, in a case–control cohort study, next-generation sequencing of 11,095 PD patients and 12,615 healthy controls revealed that neither *LRRK1* nor *LRRK2* loss-of-function variants increase or decrease the risk of developing PD [98].

In addition, a recent human study reported that carriers of the heterozygous loss-of-function LRRK2 variant who have much reduced LRRK2 protein levels show no PD-relevant phenotypes nor are they associated with any disease state [99]. Conversely, recent evidence has shown that several common *LRRK2* variants may increase the PD risk *via* alterations in LRRK2 expression level [14]. Indeed, a large-scale meta-analysis of GWAS data on disease-associated single-nucleotide polymorphisms of PD patients has identified a PD risk allele (rs76904798-T) at the *LRRK2* locus, which is associated with increased expression of LRRK2 in monocytes [100, 101]. Similarly, the cerebrospinal fluid of G2019S PD patients shows elevated levels of LRRK2 [102]. These observations are consistent with the concept that upregulated LRRK2 expression increases the PD risk in humans. In mouse models, neither overexpression of WT human LRRK2 [81, 103] nor overexpression of mouse Lrrk2 [104] in mice leads to PD-like phenotypes [105]. Such disparities between mice and humans may be attributed to the diminutive lifespan of mice that is not comparable to that of humans—particularly for neurodegenerative diseases such as PD, in which the disease symptoms develop over years. Other possible explanations for observed differences may be the slight differences in their amino acid sequences or perhaps the species-specific regulatory elements that result in species-specific LRRK2 behaviour [106].

Furthermore, the *Lrrk2* KO mouse model points to the important role of LRRK2 in peripheral tissues such as the kidneys and lungs, where it is expressed at a much higher level compared to that in other organs, including the brain. Mice devoid of LRRK2 exhibit early-onset gross morphological changes, including abnormally enlarged secondary lysosomes (phagolysosomes) in proximal tubule cells of kidney and lamellar bodies in lung type II pneumocytes [93, 94, 107, 108]. The kidneys of *Lrrk2* KO mice also show an impaired autophagy-lysosomal pathway, with accumulation of lipofuscin granules and abnormal changes in levels of autophagic markers such as LC3-II and p62 [93] and lysosomal markers LAMP1 and cathepsin D [95], implicating a regulatory role of LRRK2 in protein metabolism. Similar peripheral defects have been observed by preclinical assessments of toxicity of LRRK2 kinase inhibitors in nonhuman primates, including vacuolation in type II pneumocytes, suggesting that the observations in *Lrrk2* KO models could be associated with LRRK2 kinase inhibition [109]. The lung phenotypes seen in nonhuman primates suggest a need for attentions on potential adverse effects in the future therapeutic development of LRRK2 inhibitors for PD.

#### *LRRK2* transgenic mice

Transgenic overexpression of either WT *LRRK2 *or mutant *LRRK2 *in mice using cDNA or bacterial artificial chromosome (BAC) has highlighted the impact of pathogenic LRRK2 variants such as R1441C/G and G2019S on pathological aspects of PD. Transgenic mouse models in different studies have largely shown PD-like phenotypes, including levodopa-responsive movement defects and dopaminergic neuronal loss [36, 81, 110–112]. Transgenic overexpression of LRRK2-G2019S or LRRK2-R1441C under a human TH (tyrosine hydroxylase) promoter or a neuronal-specific CMVE/(PDGF)-β promoter leads to age-dependent neuronal loss in SNpc [36, 110–112], which is preceded by dopaminergic abnormalities, including reductions of striatal dopamine content and the number of synaptic vesicles [111, 113]. These transgenic LRRK2 mouse models consequently display defective locomotion [111, 112].

Although studies of different transgenic LRRK2 mouse models have revealed a potential role of LRRK2 in dysfunctional striatal neurotransmission and compromised dopaminergic homeostasis, they yet have mixed results. For instance, one study of mice overexpressing the G2019S or R1441G mutation showed a reduced basal level of extracellular DA and SNpc neuronal loss [36, 81, 114]. Others have shown altered striatal transmission but with the absence of DA neuronal loss [96, 115]. These raise the possibility of random insertion of transgene or the overexpression of the *LRRK2* transgene itself, potentially confounding the observed dysfunctions in different models [116], which may interfere with our understanding of native LRRK2 expressed at physiological levels. Moreover, the expression levels of *LRRK2* in different mouse lines vary from twofold to 16-fold amongst different research groups, making it challenging to make comparisons and subsequent interpretation [29, 114, 117]. Nevertheless, a more in-depth discussion of *LRRK2* transgenic mouse models can be found elsewhere [24, 118].

#### *LRRK2* KI mouse

To overcome the confounding effects of different variables within transgenic models which express supraphysiological protein levels, mouse models harbouring KI mutations of pathogenic LRRK2 variants have been developed by engineering the endogenous *LRRK2* gene to produce only a single base change [119]. These models contrast with transgenic models in which LRRK2 is expressed at supraphysiological levels, and with KO models where the protein expression is reduced or eliminated. The advantage of using mutant *LRRK2* KI mouse models lies in the maintenance of endogenous expression level of LRRK2 protein since birth, which allows exploration of the protein variants and its functional alterations in the course of disease progression over time.

Up to date, mice are the only animal models harbouring KI of pathogenic *LRRK2* mutations [24], making them appropriate animal models for exploring the pathogenesis of PD. Based on currently available *LRRK2* KI mouse models, in the following sections we review several phenotypic aspects relevant to PD (Table 1), with a particular focus on models harbouring the most frequently occurring pathogenic variants G2019S and R1441C/G, on how they potentially mimic some of the early-stage pathogenic changes in PD.

**Table 1:** Summary of *LRRK2* mutant KI mouse models with PD-relevant phenotypes

Mutation	Striatal physiology	Motor and non-motor phenotypes	Mitochondrial defects	Autophagy-lysosomal defects	Synucleinopathy/tauopathy	References
R1441C	No DA loss. Absence of AMPH-induced synaptic DA release	Absence of AMPH-induced increase in locomotor activity	NA	NA	No aggregation of α-synuclein, tau and ubiquitin	[123]
		Absence of quinpirole-induced reduction in locomotor activity				
	Aberrant synaptic PKA activity in dSPN	NA	NA	NA	NA	[124]
	Abnormal reorganization of synaptic receptor proteins in dSPN	NA	NA	NA	NA	[134]
	Reduction of evoked DA release	Impaired iSPN-associated motor learning defects	NA	NA	NA	[136]
	Decreased excitability of iSPN					
	NA	Impaired fine motor tasks and reduced locomotion	NA	NA	NA	[143]
		Reduced olfactory sensitivity				
R1441G	No DA loss. Lower DA uptake induced by reserpine	Reserpine-induced impairment in locomotor activity	NA	NA	NA	[125]
	NA	Rotenone-induced motor deficits in aged mice	Complex I deficiency after long-term treatment with rotenone	NA	NA	[142]
	NA	NA	Abnormal mitochondrial morphology. Impaired Erk-Drp1 signalling under FCCP	Impaired clearance of damaged mitochondria	NA	[159]
	NA	NA	NA	Aberrant accumulation of CMA-specific proteins in the brain: LAMP2A and HSPA8/HSC70	Increased accumulation of α-synuclein oligomers in brains of aged mice	[162]
G2019S	NA	Hyperkinetic behaviour resistant to aging-associated motor decline	NA	NA	NA	[145]
	Alterations in synaptic proteins (DAT, VMAT2)	NA	NA	NA	Increased accumulation of pSer129 α-synuclein in aged mice	[128]
	No changes in basal DA level in the striatum					
	Age-dependent reduction in basal DA level. Absence of AMPH-induced synaptic DA release		Morphological defects in mitochondria in striatum and other brain regions	NA	Increased tau puncta formation. Increased phosphorylated tau. No α-synuclein pathology	[126]
	Elevated DA and glutamate transmission in young mice that otherwise decrease with age	Reduction in exploratory behaviour with age	NA	NA	NA	[137]
	Aberrant D2-receptor responses					
	NA	NA	NA	Impaired mitophagy rescued with LRRK2 kinase inhibitor (GSK2578215A)	Impaired α-synuclein degradation in primary neurons; phenotype rescued with LRRK2 kinase inhibitor (GSK2578215A)	[160]
	NA	NA	NA	NA	Greater nigral degeneration and pSer129 aggregates in brains of aged KI mice induced by stereotactic injection of AAV2/9-A53T-α-synuclein	[183]
	Reduction of evoked DA release	NA	NA	NA	NA	[136]
	NA	NA	NA	Altered expression of autophagy proteins (LAMP2, mTOR, TFEB, GBA1)	NA	[164]
	NA	NA	NA	Impaired basal mitophagy that was rescued with LRRK2 kinase inhibitor (GSK3357679A)	NA	[165]
	NA	NA	NA	Impaired autophagosome transport	NA	[166]

Reports studying *LRRK2* KI mice carrying pathogenic *LRRK2* mutations (R1441C/G and G2019S) which show phenotypic changes in any of the five categories have been included: striatal physiology, motor and non-motor phenotypes, mitochondrial defects, autophagy-lysosomal defects and synucleinopathy/tauopathy

*NA* not applicable

### Subtle but significant PD-related phenotypes in *LRRK2* KI mice

#### Striatal neurotransmission

The major neuropathology of PD involves degeneration of dopaminergic neurons in the SNpc, which subsequently leads to loss of DA signal input in the striatum. DA is a key messenger in striatal neurotransmission that is primarily involved in the control of voluntary movement [120, 121]. Thus, DA depletion and dysregulated striatal neurotransmission lead to the debilitating motor symptoms of PD patients. Increasing evidence indicates that LRRK2 may be involved in modulating the striatal neural network. LRRK2 is expressed in both striatal axons and dendrites [103, 122] and its striatal expression level rises dramatically throughout the postnatal brain development period [11, 60], pointing to its functional relevance in the central nervous system.

In contrast to transgenic mice which exhibit PD phenotypes, the first *LRRK2* KI mouse model generated harbouring the R1441C mutation within the Roc domain displays grossly normal DA neuronal morphology and projections even after aging [93, 123]. However, upon stimulation with amphetamine (AMPH), a drug inducing synaptic DA release, R1441C KI mice lack the AMPH-induced increase in locomotor activity that is present in WT animals [123, 124], suggesting a potential defect in the DA system. Similarly, we developed mice with homozygous LRRK2 R1441G KI mutation, which exhibit perturbed DA homeostasis at as early as 3 months of age, as evidenced by significantly reduced synaptosomal DA uptake compared to that of WT controls after reserpine treatment [125]. Reserpine is a drug that irreversibly blocks vesicular monoamine transporter-2 (VMAT2). Given that the expression levels of both VMAT2 and dopamine transporter (DAT) in the striatum are similar between R1441G and WT animals, the lower DA uptake after reserpine treatment in the KI mice indicates an altered DAT function and increased susceptibility to DA depletion that may reflect the earliest presynaptic dysfunctions in PD mediated by pathogenic LRRK2. These R1441G KI mice also show greater impairment in locomotor activity induced by reserpine, with much slower motor recovery, evidencing the increased vulnerability to DA stress. These observations are important, because even without robust DA neuronal loss, the dysfunctional DA system is still a key feature of prodromal PD. While studies are available for both R1441C and R1441G KI models which have amino acid changes in the same R1441 residue (-C/G), those that explore common phenotypes in each mouse line are lacking. Moreover, the development of independent genetic lines with dissimilar genetic background in different laboratories renders the comparison of models rather difficult. Nevertheless, since both R1441C and R1441G KI mouse models show altered DA system, it is reasonable to expect that these two mutations—occurring at the same residue within the Roc domain—may share similar pathogenic mechanisms.

In accordance with mutations causing changes in the Roc-GTPase domain (R1441C/G) that mediate alterations in the striatal DA system, the G2019S variant, which causes an amino acid change in the kinase domain, also exhibits pathogenicity in DA synaptic function. The G2019S KI mice display an age-dependent reduction in basal DA levels, which is absent in WT mice [126]. Challenging the G2019S KI mice with AMPH fails to induce DA release. In fact, the amount of DA release is reduced, which may be attributed to impaired DA packaging into vesicles or impaired DA exocytosis given the normal DA metabolite levels and reverse transport of cytosolic DA. Moreover, these G2019S KI mice display an increased homovanillic acid/DA turnover ratio (DA synthesis and metabolism ratio), consistent with an enhanced striatal DA turnover observed in the early stages of sporadic PD patients [127]. The increased DA turnover is thought to be a compensatory mechanism against dopaminergic neurodegeneration, which may possibly explain the long prodromal phase before the onset of motor symptoms when the majority of DA neurons have died or are dysfunctional. In contrast to this study, another group reported no change in the basal striatal DA level in *LRRK2*-G2019S KI mice [128]. Interestingly, unlike our R1441G mice with no alterations in the levels of synaptic proteins [125], these G2019S mice exhibit an age-dependent upregulation of DAT and downregulation of VMAT2, both of which are key regulators of DA homeostasis [128]. This also suggests that different *LRRK2* mutations may exert different influences on DA synaptic proteins. The authors who produced the G2019S KI mice hypothesized that such alterations in synaptic protein levels contribute collectively to the perturbed neurotransmission *via* upregulation of DAT, which results in increased oxidative stress generated by DA autooxidation and subsequent neuronal death [129, 130]. Similar pathogenic events also occur in PD patients with reduced VMAT2 levels [131].

In addition to the role of LRRK2 in presynaptic neuronal termini, *LRRK2* mutation also affects postsynaptic function in spiny projection neurons (SPN) that populate the striatum [118]. The classical simplified model of neuronal circuitry governing movement control divides SPN into two populations based on their innervation source and projection targets: direct-pathway SPN (dSPN) "promoting movement” and indirect-pathway SPN (iSPN) “suppressing movement” [118, 132]. DA signalling has opposing effects on the activities of the two SPN populations: it increases the dSPN activity while decreasing the activities of iSPN. The balance between the two pathways receiving dopaminergic input controls motor coordination, and the net output is to promote movement. In PD, SPN loses their dopamine input from the SNpc, which causes an imbalance between the two pathways and eventually results in the characteristic motor symptoms [133]. Such perturbation in synaptic transmission in SPN has been observed in both R1441C and G2019S KI mice, an impairment mediated by abnormal reorganization of synaptic receptor proteins in dSPN [134]. In PD patients, disrupted signalling in SPN has been associated with hypokinetic symptoms [135], suggesting a role of LRRK2 in modulating SPN neurotransmission. Interestingly, LRRK2-mediated defects in SPN are stronger in R1441C than in G2019S mice, indicating increased pathogenicity conferred by the GTPase-mutant R1441C [134]. For instance, a recent study on both R1441C and G2019S KI mice has shown decreased excitability of iSPN that is associated with impaired motor learning only in R1441C mice but not in G2019S mice [136]. Similarly, R1441C KI mice show an aberrant increase in synaptic PKA activity in dSPN, which is not altered in G2019S mice [124]. It is known that the altered PKA activity results in abnormal synaptogenesis in the developing SPN, so the aberrant PKA activity in R1441C mice suggests a pathogenic role of mutant *LRRK2* in early-onset dysfunction of SPN. These may help to explain the higher PD penetrance rate in R1441C/G/H patients compared with that of G2019S carriers [73].

Dopamine D2 receptors (D2R) are postsynaptic proteins highly expressed at the postsynaptic termini of iSPN and regulate DA levels in the synaptic cleft. Both R1441C and G2019S KI mice failed to respond to quinpirole (a selective D2 receptor agonist that reduces locomotor activity), implying that the two mutant variants of LRRK2 desensitize D2R [123, 137, 138], possibly through a common pathway that is yet to be discovered. With the desensitization of D2R, the use of antagonist and agonist radioligands of dopamine D2R for functional molecular positron emission tomography (PET) imaging in human patients [139] may help reveal early D2R dysfunction before the occurrence of significant neurodegeneration prior to PD diagnosis.

Altogether, considerable evidence suggests that the majority of *LRRK2* variants alter not only presynaptic but also postsynaptic striatal neuronal activities, compromising the functions of striatal SPNs and subsequent neural outputs that control movement. Interestingly, while R1441C/G and G2019S *LRRK2* mutations confer similar presynaptic alterations in response to AMPH challenge, the reports on postsynaptic phenotypes show greater pathogenicity conferred by R1441C over G2019S. Although there is no overt neurodegeneration in SNpc of *LRRK2* KI mice, perturbations in nigro-striatal neurotransmission pathways observed in KI models may reflect early synaptic dysfunction leading to PD in humans, suggesting potential therapeutic benefits of targeting pre- and post-synaptic functions prior to the disease onset.

#### Locomotor symptoms

Mice are advantageous over other animals in modelling PD, as in mouse models degeneration in the nigrostriatal system correlates with motor impairments that can be easily assessed by various behavioural tests [140]. However, there is no PD mouse model that displays both genuine parkinsonian traits and neuropathological changes thus far. PD mouse models induced by neurotoxins such as 1-methyl-4-phenyl-1,2,3,6,-tetrahydropyridine (MPTP), rotenone and paraquat show motor dysfunction as a result of neurodegeneration induced by specific toxic insults to the SNpc. Nevertheless, these toxin-based models of PD do not fully recapitulate neuropathology of PD, as they usually lack LB pathology [141] and only display an acute neuronal loss in contrast to the progressive neurodegeneration over a long period of time in PD patients. *LRRK2* transgenic mouse models overexpressing pathogenic variants such as R1441C or G2019S exhibit DA neuronal loss, which leads to levodopa-responsive locomotor dysfunction [36, 110–112], indicating a dose effect of *LRRK2* pathogenic gene on the DA system.

In fact, behavioural studies in *LRRK2* KI mouse models have revealed a modest locomotor impairment that may correlate with subtle motor changes occurring in the prodromal phase of PD patients. For instance, we previously investigated the combined effects of gene mutation (*LRRK2*-R1441G), environment (rotenone, a pesticide and mitochondrial toxin) and aging, by oral gavage of low-dose rotenone in the R1441G KI mice for approximately half of their lifespan, and observed an increased locomotor deficit that was absent in age-matched WT mice [142]. This may serve as a novel paradigm in PD mouse model research to demonstrate an interplay of mutant *LRRK2* with other causative factors that can result in motor impairments resembling PD symptoms.

Despite the absence of severe motor dysfunction regardless of aging [143], there is a subtle but significant reduction in locomotion in the aged R1441C KI mice in more complex tasks such as the Vertical Pole test and the Ladder-and Beam Walk test. These motor tests reflect perturbation of the nigrostriatal system [144] by measuring fine changes in motor activities of mice. Interestingly, one study reported a hyperkinetic phenotype in young *LRRK2*-G2019S KI mice, which is correlated with the reported increase in DA release upon stimulation [145]. Such hyperkinetic behaviour, however, decreases with age, mirroring the subsequent reduction in the extracellular DA level [126, 137]. The authors postulated that the hyperactivity in early age may reflect a compensatory mechanism prior to nigrostriatal system impairments [145]. Nevertheless, how the different *LRRK2* mutations contribute to early changes in DA system and subsequent motor changes in both mouse models and PD patients is still unclear and requires further research.

Thus far, several lines of evidence from KI mouse models have implicated a potential role of pathogenic LRRK2 in producing subtle but progressive motor deficits with age and signs of olfactory dysfunction. Future studies investigating the LRRK2-relevant behavioural changes in KI mice may aim to use a panel of common tests on different aspects of PD motor and non-motor symptoms: bar and drag test, stepping test or pole test for measuring akinesia/bradykinesia; rotarod test for measuring balance, strength and coordination; tail-suspension test or forced swim test for assessing depression and behavioural despair [140].

#### Impaired olfaction

In addition to the compromised locomotor capabilities, impaired olfaction is one of the most common non-motor symptoms, which is present in ~ 90% of early-stage PD [146, 147]. Odour discrimination test in aged R1441C KI mice revealed reduced olfactory sensitivity compared to WT controls, reflecting that some of the early signs of diminished olfactory sensation in PD may be mediated by LRRK2 mutation [143, 147]. Olfaction has been shown to be compromised in human G2019S carriers with and without PD [148], possibly associating mutant *LRRK2* with olfactory dysfunction. However, there is limited amount of research data on olfaction of *LRRK2* KI mice. Nevertheless, the Braak staging model suggests that PD brain pathology ascends caudo-rostrally from the olfactory bulb and the dorsal motor nucleus complex of the glossopharyngeal and vagus nerves [149]. *LRRK2* KI mice are particularly useful for thorough examination of different brain regions that show early neuropathological changes associated with olfactory dysfunction, which is not possible in patients.

#### Mitochondrial dysfunction

Mitochondria play a critical role in neuronal energy homeostasis. Dysregulated mitochondrial homeostasis leads to disrupted bioenergetics in nigrostriatal DA neurons that are particularly vulnerable to dysfunction and degeneration [139, 150]. Mitochondrial dysfunction is one of the key pathogenic features of both familial and idiopathic PD [151, 152], and is associated with ATP deficiency and nigrostriatal DA neurodegeneration. Post-mortem PD brains show reduced mitochondrial Complex I activity [153, 154]. Moreover, pharmacological inhibition of mitochondrial Complex I by neurotoxins such as MPTP leads to selective degeneration of DA neurons in mice [155]. Genetic studies also revealed that mutations of various mitochondrial genes such *PRKN*, *PINK1* and *DJ-1* are causative factors for PD [156], further suggesting mitochondrial dysfunction as one of the pathogenic events in PD.

LRRK2 under physiological conditions is mainly localized in cytoplasm, but is also found in nucleus and mitochondria. Its pathogenic mutations have been increasingly linked with mitochondrial dysfunction in PD [151, 157]. In heterozygous and homozygous *LRRK2*-G2019S KI models, mitochondrial morphological abnormalities in aged striatum occur in a gene dose- and age-dependent manner [126]. The abnormal shape resembles beads-on-the-string morphology, which possibly denotes defects in fission and fusion [158], suggesting that the LRRK2 variant may impair mitochondrial fission—a key process for maintaining healthy mitochondrial network. Interestingly, alterations in mitochondrial shape are not limited to striatum but also occur in other brain regions such as cortex and to a lesser extent the hippocampus, indicating that the LRRK2 variant-driven changes in mitochondrial morphology may be tissue-specific. Similarly, we recently reported abnormal mitochondrial morphology denoting impaired fission and disrupted clearance of damaged mitochondria in the striatum of aged *LRRK2*-R1441G KI mice [159]. Most importantly, our previous work also revealed that the R1441G KI mice receiving low doses of rotenone for over half of their lifespan to mimic the effect of environmental influence over time in PD, show Complex I deficiency [125]. Such defects were absent in the WT controls receiving the same treatment. Therefore, the study results suggest combined effects of LRRK2 variant and other risk factors—aging and environment toxins—that result in mitochondrial dysfunction, leading to PD.

Collectively, *LRRK2* KI mice, despite having different mutations, display pathologies on mitochondrial function and morphology *in vivo*, suggesting the importance of physiological LRRK2 in maintaining mitochondria homeostasis and DA neuronal survival. Therefore, targeting pathogenic LRRK2 to mitigate mitochondrial dysfunction appears to be a reasonable option for delaying neurodegeneration and thereby modifying the course of the disease. This also highlights *LRRK2* KI mice as a suitable *LRRK2*-PD model system for studying mitochondrial dysfunction in PD. Further work is required to pinpoint the exact mechanism of LRRK2-mediated defects, including whether or not the kinase hyperactivity is involved.

#### Defects in autophagy and endo-lysosomal pathways

An orchestrated network of endo-lysosomal and autophagic pathways is crucial for maintaining protein homeostasis and turnover in damaged organelles in the brain. A defective network in humans may trigger pathologies similar to PD. Evidence suggests that dysfunction of these pathways is associated with LRRK2 mutations, such as abnormally high lysosomal pH in *LRRK2-*G2019S KI mice-derived cortical neurons, impaired clearance of autophagosomes and defective autophagosome-lysosome fusion in R1441C-overexpressing neurons [36, 160, 161]. Furthermore, we have shown that aged mutant *LRRK2*-R1441G KI mice display accumulation of LAMP2A and HSPA8/HSC70 proteins in the striatum [162], suggesting defective chaperone-mediated autophagy, a specific route for clearance of various proteins such as α-synuclein *via* LAMP2A-mediated lysosomal degradation [162, 163]. We have also demonstrated impaired clearance of defective mitochondria in *LRRK2*-R1441G KI mouse embryonic fibroblasts (MEFs), associated with abnormal clustering of mitochondria and impaired DRP1-ERK signalling under mitochondrial stress [159]. Similarly, *LRRK2*-G2019S KI mice show accumulation of LAMP2A and downregulation of a number of key proteins in the autophagy-lysosome pathway, including LAMP2, mTOR, TFEB and GBA1 [164], further confirming the involvement of LRRK2 in autophagic pathways.

Although the detailed pathogenic mechanisms of *LRRK2* variants in PD are still unclear, hyperactive kinase activity appears to be an important disease marker of both LRRK2 and non-LRRK2 PD. *LRRK2*-G2019S KI mice with hyperactive LRRK2 show impaired mitophagy, which is rescued with therapeutic LRRK2 kinase inhibition in MEFs derived from the same mouse as a proof of concept [160, 165]. Another study demonstrated that the hyper-kinase activity in G2019S KI mice impairs neuronal autophagy by specifically modulating axonal transport of the autophagosomes in a kinase-dependent manner [166]. Such impairment is associated with an increased level of motor adaptor protein JNK interacting protein 4 recruited on the autophagosomes, and hyperactive variant of LRRK2 was proposed to abnormally activate the motor protein kinesin, which delays autophagosome transport, eventually resulting in an abnormal accumulation of protein aggregates. This is also reflected by the accumulation of α-synuclein deposits in the Lewy neurites of neuronal axons where autophagosomal transport actively occurs [167]. This demonstrates the suitability of mutant *LRRK2* KI mouse model in recapitulating the axonal pathology preceding axonal degeneration in PD [168]. Overall, different *LRRK2* KI mouse studies have provided robust evidence of impaired autophagy-lysosomal system, reflecting similar defects in PD.

#### Synucleinopathy

α-Synucleinopathies are a collection of diseases characterized by abnormal accumulation of α-synuclein aggregates in neurons, nerve fibres or glial cells, including PD, dementia with Lewy bodies, multiple system atrophy and rare conditions including inherited metabolic disorders, e.g., Gaucher's disease [169]. In PD, the collapse of intracellular transport and degradation system for damaged organelles, together with the spontaneous aggregation of certain proteins, leads to accumulation of different pathogenic protein entities formed primarily from α-synuclein and tau [170, 171]. Autopsy examinations have revealed that the majority of *LRRK2*-PD patients present with LB-like α-synuclein pathology. Immunohistochemical analysis of brain samples of PD patients revealed that a significant portion of α-synuclein-positive LBs contained LRRK2 [172], further supporting the association between LRRK2 and α-synuclein. Approximately 24% of LBs in idiopathic patients were LRRK2-positive, whereas in confirmed *LRRK2*-PD patients with G2019S mutation, the proportion of LRRK2-reactive LBs increased to 50% [173], implicating a pathogenic role of LRRK2 in synucleinopathies. α-Synuclein is known to interact with some of the endocytic kinase substrates of LRRK2, such as Rab3a, Rab8 and Rab35 [174, 175], suggesting that LRRK2 and α-synuclein share similar pathogenic pathways *via* pathogenic hyper-phosphorylation of these Rabs, which may lead to mishandling of α-synuclein.

*LRRK2* KI models have been widely used to study the potential interaction between LRRK2 and α-synuclein in the development of synucleinopathy in PD. Varying severities of α-synuclein pathology have been reported in *LRRK2* KI mice, possibly due to the intrinsic variability of protein aggregation. For example, *LRRK2*-R1441C KI mice do not show increased aggregation of proteins including α-synuclein, tau and ubiquitin [123]. Similarly, no α-synuclein or tau pathology is found in the striatum of *LRRK2*-R1441G KI mice [125]. However, we have recently shown that the striatum of *LRRK2*-R1441G KI mice displays more severe age-dependent accumulation of α-synuclein oligomers than that in age-matched WT littermates [162]. These oligomers represent the toxic species that induce neuronal death [176].

In contrast to R1441C/G KI mice [123], the mice with G2019S mutation show more varied pathologies. One study showed that the *LRRK2*-G2019S mice have increased tau phosphorylation correlated with positive puncta of phosphorylated tau, but without α-synuclein pathology [126], while others have reported age-dependent elevations of phosphorylated α-synuclein and α-synuclein inclusions in the striatum of G2019S KI mice [128]. A potential modulatory role of LRRK2 in fibril-induced α-synuclein aggregation has been shown by comparing primary cortical neuronal cultures derived from G2019S KI mice with those from *Lrrk2* KO mice [177]. The G2019S KI neurons show increased vulnerability to α-synuclein accumulation triggered by fibrils, whereas the *LRRK2 *KO neurons showed robust resistance to aggregation [177].

While the molecular interaction between LRRK2 and α-synuclein is still unresolved, studies have investigated whether the pathogenicity is mediated *via* the hyper-kinase activity conferred by the pathogenic *LRRK2* mutations. Interestingly, primary neurons derived from G2019S KI mice have impaired α-synuclein metabolism, which is reversed by pharmacological inhibition of LRRK2 kinase activity [160]. Similarly, multiple human induced pluripotent stem cell-derived neural lines derived from fibroblasts of G2019S PD patients show increased accumulation of α-synuclein, which can be reduced with LRRK2 inhibitors [178]. The authors postulated that the increased accumulation of α-synuclein could be attributed to mutant LRRK2 that negatively affects the autophagy-lysosomal system. Administration of a LRRK2 kinase inhibitor into α-synuclein transgenic mice also significantly decreases the α-synuclein accumulation in several regions of the brain, including neocortex and striatum [175], further confirming the role of LRRK2 hyperactivity in synucleinopathy.

Although a growing body of evidence suggests that the α-synuclein accumulation may be mediated by the hyper-kinase activity of mutant LRRK2, contradictory results have been reported. Hippocampal neurons derived from a transgenic BAC mouse expressing the G2019S variant protein show a mild but reversible elevation in α-synuclein pathology [179]. The same neuronal cultures treated with α-synuclein pre-formed fibrils which resulted in the development of α-synuclein pathology showed no response to LRRK2 kinase inhibitor treatment. A more recent study conducted by the same group showed that neurons derived from *LRRK2*-G2019S transgenic mice exhibit a robust tau pathology that is also insensitive to the treatment with a LRRK2 kinase inhibitor [180], adding to the complexity of the interaction between LRRK2 and pathological proteins of PD. It should be noted, however, that α-synuclein, being a dynamic protein in nature, may develop into pathological forms *via* different mechanisms, which may or may not interact with LRRK2 for further pathogenicity.

In mouse models, *LRRK2* mutations alone have produced mild-to-no signs of severe synucleinopathy. The pathogenicity of LRRK2 variants may be enhanced in the presence of other factors such as aging and PD-causing α-synuclein mutations. For instance, one study performed a stereotactic injection of AAV2/9 vectors expressing a human A53T α-synuclein (a pathogenic PD-causing variant which is aggregation-prone) into the striatum of both young and aged *LRRK2*-G2019S KI mice and WT mice [181, 182]. This resulted in greater nigral degeneration and pSer129 α-synuclein aggregates in the aged *LRRK2*-G2019S KI mice compared to their age-matched WT littermate controls [183]. These demonstrated an interplay between pathogenic LRRK2, mutant α-synuclein and aging in accelerating development of α-synuclein pathology implicated in PD. Similarly, A53T α-synuclein transgenic mice overexpressing *LRRK2* display degeneration of DA neurons and aggregation of α-synuclein [96], which are absent in transgenic mice overexpressing LRRK2 alone. These findings clearly show that cross-breeding of *LRRK2* KI mice with mice carrying other genetic risks facilitates a deeper elucidation of the interplay of LRRK2 with other PD factors that may speed up the development of α-synuclein pathology. Therefore, *LRRK2* KI mice represent a good *in vivo* model to observe different stages of synucleinopathy under the co-influence of LRRK2 and secondary factors such as aging and genetics.

### Limitations of *LRRK2*-based mouse models

*LRRK2-*based mouse models have been proven valuable for elucidating some of the LRRK2-mediated pathogenic effects and how LRRK2 interacts with other risk factors to modulate PD pathology. With highly similar genetic make-up to humans but a much shorter lifespan, PD mouse models enable fast and thorough investigation of the role of various PD risk genes and their disease mechanisms, as well as the interactions among genetic risks, aging and environmental factors. Furthermore, as in many other neurodegenerative diseases, natural aging is a key determinant of the onset and progression of PD. The accumulation of pathological proteins in the affected brain regions is a gradual process that may take years, if not decades in humans, until exceeding a threshold when disease symptoms are triggered. It is possible that the short lifespan of rodents may be technically insufficient to fully capture the absolute rate of pathological protein accumulation that occurs in human brains, which may be one of the major shortcomings in modelling aging-related neurodegenerative diseases. Moreover, despite that murine *Lrrk2* gene shares 88% sequence-similarity with human *LRRK2*, mouse Lrrk2 may not recapitulate the full spectrum of biochemical activity and pathogenic effects of human LRRK2. The fundamental differences in the development and function of mouse and human brains call for cautions when interpreting behavioural symptoms in mice, particularly when analyzing regression of cognitive function and language involved in human neurodegenerative diseases [184].

Another limitation is that no animal model can replicate all of the characteristic clinical and neuropathological symptoms of PD, including motor dysfunction, dopaminergic neurodegeneration and synucleinopathy. The resulting phenotypes of *LRRK2* KI mice are usually mild, and no KI models thus far have displayed both characteristic DA neuronal loss and severe motor deficits of PD, even at an advanced age [125, 126, 143]. The various mouse lines, with their differing genetic backgrounds, developed by different labs, and the gap of knowledge on PD pathogenesis, contribute to limitations of KI mouse models. Nevertheless, it should be emphasized that PD is a complex disease affected by interactions of multiple risk factors, in which some can still be efficiently captured in mouse models.

### Therapeutics targeting LRRK2 in PD

Pathogenic missense mutations in *LRRK2* result in abnormal enzymatic kinase and GTPase activities, which can be a target for disease modification. With the increased rate of *LRRK2* mutations found in sporadic PD cases and the indistinguishable neuropathology in both *LRRK2*-PD and sporadic PD [14, 91], LRRK2 is regarded as a promising therapeutic target against a wide spectrum of this disease. The earliest development targeting LRRK2 is the LRRK2 kinase inhibitors, as a majority of pathogenic LRRK2 variants possess upregulated kinase activity by 2-to-3 folds [66]. World-wide efforts have produced several chemically distinct classes of LRRK2 kinase inhibitors as effective pharmaceutical tools in research, including MLi-2 [185] and GNE-7915 [186], preceding the actively on-going human clinical trials on small LRRK2 kinase inhibitors BIIB122/DNL151 and DNL201 conducted by Denali Therapeutics (DNL151, ClinicalTrials.gov Identifier: NCT04056689 and DNL201, ClinicalTrials.gov Identifier: NCT03710707). Recent interim updates revealed on-target efficacy and safety of both inhibitors. Thus, new trials involving *LRRK2*-PD and sporadic PD patients are being carried out. Several biomarkers of LRRK2 kinase substrates have been proposed to reflect the on-target efficacy of these LRRK2 kinase inhibitors *in vivo*, including Rab10 and Rab12 [44]. Interestingly, Rab10 and Rab12, which are both well-established LRRK2 substrates, have shown different sensitivities in different tissues of mice. Rab10 shows higher sensitivity in the peripheral organs such as lungs and kidneys, while Rab12 better reflects the brain LRRK2 activity [187]. The extent of Rab10 phosphorylation also differs among distinct *LRRK2* mutations. Mice carrying R1441C show higher phosphorylation of Rab10 in kidneys and lungs compared with G2019S mice [188]. Therefore, it is critical that appropriate biomarkers are used in different models for a more reliable readout of LRRK2 activity.

It should be noted that all of the developed kinase inhibitors, including Denali Therapeutics, are type I kinase inhibitors [189] that achieve kinase inhibition without rescuing the pathogenic, closed conformation of variant LRRK2 [190]. One study used LRRK2 fragment containing four C-terminal domains to show that such a closed conformation of kinase promotes microtubule binding, while the open, catalytically-inactive one prevents it [190]. Another study showed that the overexpressed pathogenic I2020T LRRK2 preferentially adopts a closed conformation, which is associated with increased binding to microtubules, possibly leading to dysregulated intracellular transport [82, 191]. This is also in line with the finding that overexpression of *LRRK2* variants such as R1441C/G, Y1699C and I2020T causes increased microtubule binding, which is associated with aberrant filament formation in cells [192]. Type II kinase inhibitors inhibit LRRK2 kinase activity while maintaining an open LRRK2 conformation, therefore complementing the limitation of type I inhibitors. Recently, several type II kinase inhibitors, such as GZD-824 [190] and Rebastinib [191], were found to inhibit LRRK2 kinase activity, as they suppressed Rab10 phosphorylation while preventing aberrant LRRK2 binding to microtubule filaments [193]. However, both GZD-824 and Rebastinib were initially developed for treatments of leukemia [194], and they show poor selectivity for LRRK2 [193]. Therefore, development of novel type II inhibitors with high selectivity towards LRRK2 remains a big challenge for advancement of LRRK2 kinase inhibitors in PD therapeutics. Another major challenge in LRRK2 inhibitor development is the adverse side effects on peripheral organs of non-human primates despite attaining on-target LRRK2 inhibition [108, 109]. This emphasizes the importance of proper dosing of LRRK2 inhibitors to achieve efficacy in brain without damaging peripheral organs. The use of *LRRK2* KI model remains a fast and practical approach for initial testing of drug efficacy and safety prior to clinical trials in humans.

To address concerns of adverse effects due to excessive LRRK2 inhibition, RNA interference approach has been used to target only R1441G and R1441C alleles without affecting WT *LRRK2* in 293FT cells [195]. If successful, this technique could benefit such familial PD patients and carriers of heterozygous R1441C/G variants without triggering adverse effects from targeting WT LRRK2. Similarly, others have developed a G2019S-selective kinase inhibitor EB-42168, which achieved 90% pSer935-LRRK2 inhibition in blood of homozygous G2019S patients, 36% in heterozygous G2019S carriers, and only 5% in healthy control’s blood [196], demonstrating the potential use of precision medicine specifically for G2019S PD patients. Moreover, other types of therapeutics have been developed, such as antisense oligonucleotides (ASOs), which reduce Lrrk2 levels only in brains of transgenic mice without altering the protein level in other organs, thereby minimizing unnecessary inhibitory effects on peripheral tissues [197]. Such long-term ASO treatment reduces DA neuronal loss and subsequent motor defects in a transgenic mouse model [197]. Thus far, numerous therapeutic strategies against LRRK2 have shown promising results but require further optimization until their safety and efficacy can be justified before human clinical trials.

## Conclusion and future directions

LRRK2 is involved in various cellular pathways and neurotransmission, mutations of which can increase the susceptibility to PD. Studies using mutant KI mouse models with endogenous level and pattern of LRRK2 expression, have revealed dysfunction in several key pathological aspects of PD, mediated by pathogenic variants of *LRRK2*. Despite the relevance and usefulness of *LRRK2* KI mouse models in understanding disease pathogenesis, it must be emphasized that PD is a multifactorial disease. The interplay of different risk factors such as aging, genetic susceptibility and environmental toxins, which may be necessary to recapitulate the core features of PD, was still lacking in many studies [198]. One of our previous long-term studies successfully combined the effects of aging, environmental stress and genetic susceptibility as a viable research approach to demonstrate life-long pathogenic progression and locomotor dysfunction relevant to PD [142]. Importantly, LRRK2 remains a promising target for modulating the disease. An increasing number of clinical trials on LRRK2-targeting therapies are under development. Given the concerns of experimentation in non-human primates with respect to research cost, duration and limited number of subjects, *LRRK2* mouse models remain an invaluable, initial *in vivo* experimental platform for understanding disease pathogenesis and for evaluation of novel therapeutics in terms of bioavailability, safety and on-target tissue specificity to PD.

## Data Availability

Not applicable.

## Abbreviations

AMPH: Amphetamine
BAC: Bacterial artificial chromosome
D2R: Dopamine D2 receptor
DA: Dopamine
DAT: Dopamine transporter
GWAS: Genome-wide association studies
KI: Knock-in
KO: Knockout
LRRK2: Leucine-rich repeat kinase-2
MEF: Mouse embryonic fibroblasts
MPTP: 1-Methyl-4-phenyl-1,2,3,6,-tetrahydropyridine
PD: Parkinson’s disease
PKA: Protein kinase A
Roc-COR: Ras of complex-carboxy terminal of Roc
SNpc: Substantia nigra pars compacta
VMAT2: Vesicular monoamine transporter-2
